# A new sex-specific underlying mechanism for female schizophrenia: accelerated skewed X chromosome inactivation

**DOI:** 10.1186/s13293-020-00315-6

**Published:** 2020-07-17

**Authors:** Xinzhu Zhang, Yuhong Li, Lei Ma, Guofu Zhang, Min Liu, Chuanyue Wang, Yi Zheng, Rena Li

**Affiliations:** 1 Beijing Institute of Brain Disorders, Laboratory of Brain Disorders, Ministry of Science and Technology, Collaborative Innovation Center for Brain Disorders, Capital Medical University, Beijing, China; 2 The National Clinical Research Center for Mental Disorders & Beijing Key Laboratory of Mental Disorders, Beijing Anding Hospital, Capital Medical University, Beijing, China

**Keywords:** X chromosome inactivation, Schizophrenia, Onset age, PANSS, Skewing

## Abstract

**Background:**

X chromosome inactivation (XCI) is the mechanism by which the X-linked gene dosage is adjusted between the sexes. Evidence shows that many sex-specific diseases have their basis in X chromosome biology. While female schizophrenia patients often have a delayed age of disease onset and clinical phenotypes that are different from those of males, it is unknown whether the sex differences in schizophrenia are associated with X-linked gene dosage and the choice of X chromosome silencing in female cells. Previous studies demonstrated that sex chromosome aneuploidies may be related to the pathogeneses of some psychiatric diseases. Here, we examined the changes in skewed XCI in patients with schizophrenia.

**Methods:**

A total of 109 female schizophrenia (SCZ) patients and 80 age- and sex-matched healthy controls (CNTLs) were included in this study. We evaluated clinical features including disease onset age, disease duration, clinical symptoms by the Positive and Negative Syndrome Scale (PANSS) and antipsychotic treatment dosages. The XCI skewing patterns were analyzed by the methylation profile of the *HUMARA* gene found in DNA isolated from SCZ patient and CNTL leukocytes in the three age groups.

**Results:**

First, we found that the frequency of skewed XCI in SCZ patients was 4 times more than that in the age- and sex-matched CNTLs (p < 0.01). Second, we found an earlier onset of severe XCI skewing in the SCZ patients than in CNTLs. Third, we demonstrated a close relationship between the severity of skewed XCI and schizophrenic symptoms (PANSS score ≥ 90) as well as the age of disease onset. Fourth, we demonstrated that the skewed XCI in SCZ patients was not transmitted from the patients’ mothers.

**Limitations:**

The XCI skewing pattern might differ depending on tissues or organs. Although this is the first study to explore skewed XCI in SCZ, in the future, samples from different tissues or cells in SCZ patients might be important for understanding the impact of skewed XCI in this disease.

**Conclusion:**

Our study, for the first time, investigated skewed XCI in female SCZ patients and presented a potential mechanism for the sex differences in SCZ. Our data also suggested that XCI might be a potential target for the development of female-specific interventions for SCZ.

## Introduction

Schizophrenia (SCZ) is a chronic brain disorder with great physical morbidity and high mortality [1]. Substantial evidence shows that SCZ occurs more frequently in men than in women and that there is a sex difference in SCZ in clinical symptoms, cognitive function, onset age, and even treatment response [2, 3]. Whether the X chromosome plays any role in the sex-specific differences in SCZ is unknown. Many genes associated with psychiatric diseases, including SCZ, are located on the X chromosome [4–6]. These genes are proven participants in the basic differentiation process of neurons, encoding proteins involved in synaptic transmission [7–9]. In particular, the importance of X-linked genes is their specific impacts on the development of the amygdala, which is associated with SCZ [10, 11]. Therefore, X chromosome abnormality may be a new target for sex-specific risk of schizophrenia [4].

There are two major aspects regarding X chromosome function: X chromosome aneuploidies and X chromosome inactivation (XCI) [12, 13]. The relationship between X chromosome aneuploidies and SCZ has been widely reported by different groups, such as a higher frequency of having extra copies of the X chromosome or an XO karyotype in female SCZ patients than in the general female population [14–16], and some reports have shown the abnormal passage of the Y chromosome in male SCZ patients [17, 18]. However, studies of XCI and psychiatric diseases are limited. Females have two X chromosomes, and one is activated while the other is inactivated to maintain an equal dosage of X-linked genes with males. XCI is initiated by the transcription of *XIST*, a 17 kb, alternatively spliced long noncoding RNA mapped to Xq13.2 and exclusively expressed on the inactive X (Xi) chromosome [19]. Once transcribed, *XIST* molecules spread in cis along the X chromosome [20], inducing progressive epigenetic silencing through the recruitment of chromatin remodeling enzymatic complexes, which impose repressive histone and DNA changes on the Xi chromosome [21, 22]. Within each cell, the parental X chromosome selected for inactivation seems to occur at random, and the Xi chromosome is mitotically inherited by future somatic daughter cells. In normal situations, the initial choice of X chromosome (maternal or paternal) silencing is random but stably inherited. Skewing is defined as when a deviation from equal (50%) XCI of each parental allele occurs [23]. The most common criterion for “skewed” XCI has been defined as XCI from the same allele in 75% or 80% of cells [24–26], while very skewed XCI is defined as 90% of cells with same-allele XCI [27]. While skewed XCI has been found to be associated with immune disease, thyroid disease and cancer [28–30], recent studies have found that the frequency of skewed XCI is related to aging, with even higher skewing in older patients with Alzheimer’s disease or Parkinson’s disease [27, 31]. In psychiatric disease studies, X-linked intellectual disability has been reported in association with skewed XCI as well as X chromosome gene variants in these patients, for example, in *MECP2*, *DDX3X*, *SMC1A* [5, 32]. A higher frequency of skewed XCI was also reported in children with autism than in age-matched healthy controls [33]. Together, skewed XCI may have important impact on the sex-specific pathogenesis of psychiatric diseases.

To explore the linkage between XCI skewing and SCZ, the aims of the present study were as follows: (1) to investigate XCI skewing in patients with SCZ or major depressive disorder (MDD) and age-matched controls (CNTLs); (2) to identify the association between clinical symptom severity and the degree of skewed XCI in SCZ patients; (3) to study age-related changes in XCI skewing in young, middle-aged and elderly female SCZ patients and compare them with those of matched CNTLs; and (4) to explore whether skewed XCI in SCZ children is transmitted from parents.

## Methods

### Subjects

A total of 227 female subjects aged with a range from 8 to 77 years, were enrolled in this project. The study population consisted of 109 SCZ patients, 38 MDD patients, and 80 CNTLs. To investigate the relationship between age (age of sampling) and XCI pattern, all SCZ patients and age-matched CNTLs were subgrouped by age as children (age ≤ 18), adults (age 18-35), and elderly individuals (age ≥ 50), as shown in Table 1. The MDD patients, who served as psychiatric disease controls for the SCZ patients, were also subgrouped by age as adults (age 18-35) and elderly individuals (age ≥ 50). The SCZ and MDD patients were diagnosed by at least two experienced psychiatrists according to the criteria of the Diagnostic and Statistical Manual of Mental Disorders, fourth or fifth edition (DSM-IV or DSM-V, respectively). CNTL subjects were in general good health and without a history of psychiatric disorders or neurological disease. This study was approved by the ethics committee of Beijing Anding Hospital, Capital Medical University. Psychiatric symptoms in SCZ patients were evaluated by the Positive and Negative Syndrome Scale (PANSS) [34]. Based on the severity of clinical symptoms, the SCZ patients were further subdivided into more severe (PANSS scores ≥ 90) and less severe (PANSS scores < 90) phenotypes in some experiments. Depressive symptoms were assessed with the 17-item Hamilton Depression Rating Scale (HAMD-17) [35]. Antipsychotic treatment dosages were converted to olanzapine (mg) by “DDD” (dose equivalents based on defined daily doses) in SCZ (13.42 ± 0.84 mg/day) and MDD patients only treated by escitalopram (14.21 ± 0.87 ma/day). To examine the genetic impact of XCI, parents of 14 pediatric SCZ patients were also enrolled in this study.

**Table 1:** Genotypic frequencies of subjects enrolled in the study and the prevalence of skewed X chromosome inactivation

Group 1	Group 2	Total no. of women enrolled in the study	Age (mean and standard deviation)	No. of women heterozygous at the HUMARA locus	No. of individuals with skewing ≥75%	% of skewing ≥75%	OR	CI	*p*-value	No. of individuals with skewing ≥80%	% of skewing ≥80%	OR	CI	*p*-value
CNTL	SUM	80	28.61±14.6	80	10	12.5				5	6.25			
	Child	20	13.35±4.65	20	1	5				0	0			
	Adult	47	27.23±3.67	47	6	12.2				3	6.1			
	Elderly	13	57.07±7.27	13	3	23				2	15.3			
SCZ	SUM	109	27.7±15.00	101	29	28.7	2.8	1.2-6.2	p=0.01	20	19.8	3.7	1.3-10.3	p=0.009
	Child	45	15.12±2.14	39	6	15.3	3.4	0.4-3.8	p=0.404	3	7.6	1.5	0.8-1.0	p=0.156
	Adult	48	27.89±4.39	46	18	39	4.3	1.3-12	p=0.005	14	30	6.4	1.7-24	P=0.006
	Elderly	16	57.81±7.23	16	5	31	1.5	0.3-8.0	p=0.67	3	19	1.2	0.18-9.0	p=0.81
MDD	SUM	38	38.31±15.3	38	7	18.4	1.8	0.6-5	p=0.392	6	15.7	1.7	0.4-6.9	p=0.1
	Adult	22	25.86±4.15	22	4	18	0.7	0.4-6.0	p=0.714	4	18	3.2	0.6-16	p=0.192
	Elderly	16	55.44±4.37	16	3	19	0.8	0.1-4.6	p=1	2	13	1.1	0.16-8.1	p=1

Considered skewed if the inactivation of the predominant allele was ≥75% or 80%, ORs and values for patients vs the age-matched heathy control group. SCZ vs CNTL, p<0.05*; age group SCZ vs CNTL, p<0.0167 * or age group MDD vs CNTL, p<0.025* by Fisher’s exact test or the chi-squared test. OR=odds ratio CI= confidence interval

### DNA extraction

Whole-blood samples from subjects were centrifuged at a speed of 3000 rpm for 10 min to extract the intermediate layer and separate the leukocytes. Genomic DNA was extracted from leukocytes using a genomic DNA purification kit (Promega, Madison, WI, USA). Leukocytes were processed for DNA isolation as follows: 200 μl of PBMCs was digested with proteinase K in cell lysis buffer at 56°C for 10 min, and then the genomic DNA was extracted by a salting-out procedure and dissolved in nuclease-free water according to the standard manufacturer’s protocol.

### Analysis of skewed XCI

The identification of the two X chromosomes depends on the polymorphism display. The most commonly used polymorphism site is the short-tandem repeat (STR) of CAG at exon 1 of the Xq13 *HUMARA* gene, the human androgen receptor locus for XCI pattern experiments, as previously described [26, 28]. The methylation profile of the *HUMARA* gene, located on the X chromosome, was used to determine XCI ratios as previously described [24]. Duplicated DNA aliquots from each sample were digested with restriction enzymes: HpaII, a methylation-sensitive enzyme, and the methylation-insensitive enzyme RsaI, which was used as a control for input DNA [36]. Fifty nanograms of DNA was digested at 37 °C for 2 h with 1 μl of HpaII (New England Biolabs) and 1 μl of RsaI (New England Biolabs). For each sample, a control sample (no HpaII) was similarly prepared using only 1 μl of RsaI enzyme. Male DNA was used as a negative-control sample. The sequences of the primers used were as follows: forward 5′-TCCAGAATCTGTTCCAG AGCGTGC-3′, labeled with 5′-FAM, and reverse 5′-GCTGTGAAGGTTGCTGTTCC TCAT-3′ [24]. The samples were amplified for 35 cycles, including 30 s at 95°C, 60 s at 55°C, and 60 s at 72°C with an initial denaturation at 95°C for 10 min. The PCR products were separated by an ABI3500 Genetic Analyzer (ABI, Thermo Fisher Scientific). The size of the PCR product from each allele was analyzed by GeneMapper v4.1 for the quantification of peak height. The evaluation of XCI skewing was performed as previously described [25, 37].

$$ \mathrm{Psup}=1-\frac{\left\{\frac{\frac{\mathrm{A}}{\mathrm{A}+\mathrm{a}}}{\frac{A^{\prime }}{{\mathrm{A}}^{\prime }+\mathrm{a}^{\prime }}}\right\}}{\left\{\frac{\frac{\mathrm{A}}{\mathrm{A}+\mathrm{a}}}{\ \frac{A^{\prime }}{{\mathrm{A}}^{\prime }+\mathrm{a}^{\prime }}}+\frac{\frac{\mathrm{a}}{\mathrm{A}+\mathrm{a}}}{\frac{a^{\prime }}{{\mathrm{A}}^{\prime }+\mathrm{a}^{\prime }}}\right\}} $$

The *P*_sup_ score indicates the proportion of cells with the longer *HUMARA* allele on the active X chromosome. A and A′ are the peak heights of the longer *HUMARA* allele from the digested and undigested samples, respectively. a and a′ are the peak heights of the shorter *HUMARA* allele from the digested and undigested samples, respectively.

For the prevalence of skewing (% of skewing), that is, the prevalence of skewed XCI in each group, the most common cut-offs for calculating the prevalence of XCI skewing (% of skewing) are ≥ 75:25% [25] and ≥ 80:20% [38]. The degree of skewing (DS) designates the percentage of the preferentially active allele and does not take into account the direction of skewing but only the degree of deviation from a 50% XCI pattern. DS is calculated using the formula |*P*_sup_ − 0.5| and represents a continuous variable that ranges between 0 and 50%, where 0% indicates a random X inactivation pattern and 50% indicates a completely skewed inactivation pattern. The relationship between the XCI skewing from the mother of the SCZ patient and the XCI skewing from the patient themselves was calculated by the quantitative assessment of XCI ratio transmission as described before [25]. *P*_mat_ and *P*_trans_ are calculated the same way as *P*_sup_, except that A indicated the HUMARA allele shared between mother and daughter.

### Statistical analysis

Continuous variables are described as the mean ± Standard Error of Mean (SEM), and the categorical variables are presented as the number (the percentage). The distribution of continuous variables was decided by the Kolmogorov-Smirnov test followed by the Mann-Whitney *U* test for nonparametric data comparison, Fisher’s exact test or the chi-squared test. *P* values below 0.05 were considered statistically significant, with Bonferroni’s correction for each age group analyzed (*p* < 0.0167, 0.05 divided by 3 for each age group). The associations between patient age and skewed XCI in the schizophrenia were evaluated using Spearman’s correlation and simple linear regression analysis. The univariate and multivariate logistic regression analyses were performed to calculate the odds ratio (OR) of patient age in schizophrenia with an increase of 10 and the corresponding 95% CI in discriminating the presence of severely skewed XCI (≥ 80%) in schizophrenia before and after adjusting for confounding factors, including PANSS sum score and medication. Patient age and the age of onset were compared by paired-sample tests. Data analyses were performed using SPSS version 23.0 (IBM Corp, Armonk, NY, USA).

## Results

### Higher frequency of severe XCI skewing in schizophrenia patients

**Fig. 1:**
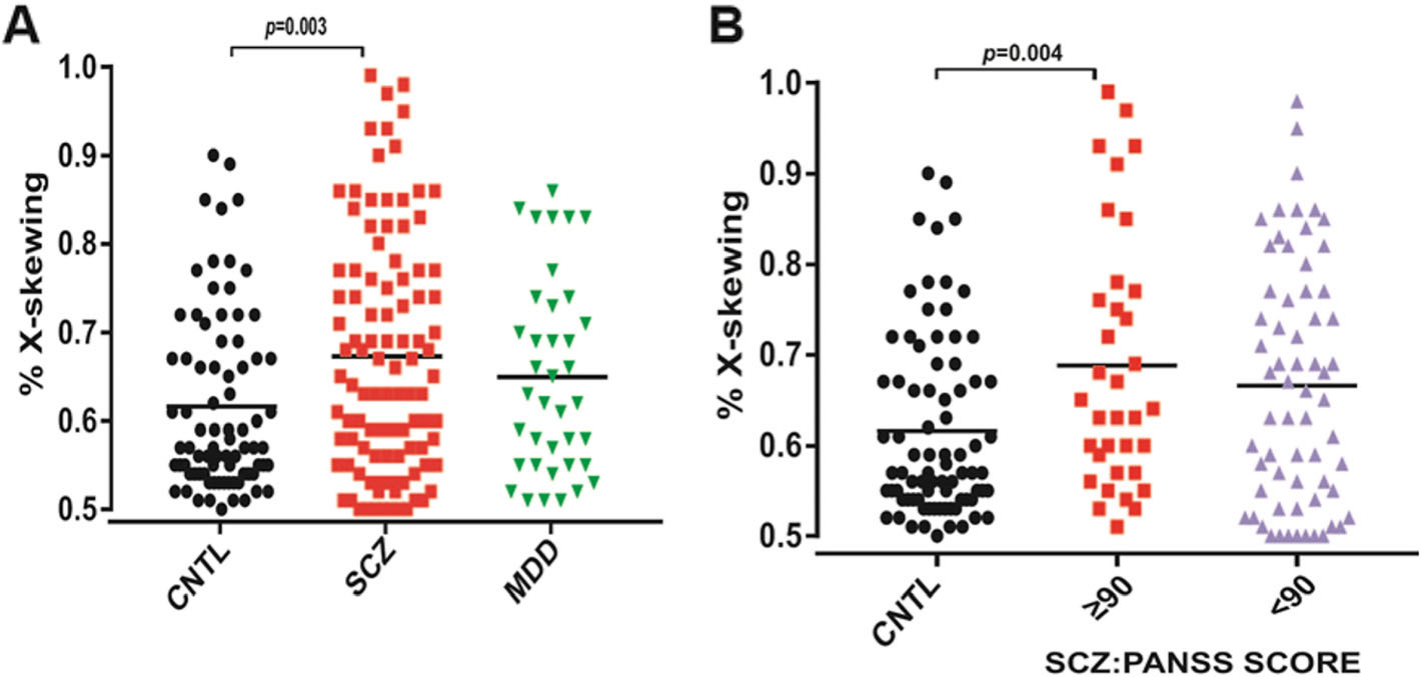
The horizontal lines represent the median, and significant differences are highlighted (Mann-Whitney *U* test). **a** Higher frequency of severe XCI skewing in schizophrenia patients. The results of female healthy controls (CNTLs) (*n* = 80), SCZ patients (*n* = 101), and MDD patients (*n* = 38) are shown. CNTL vs SCZ *p* = 0.003, CNTL vs MDD *p* = 0.363. **b** Higher frequency of severe XCI skewing in patients with severe symptoms of schizophrenia. The results of female healthy controls (CNTLs) (*n* = 80), SCZ: PANSS score ≥ 90 (*n* = 35), and SCZ: PANSS score < 90 (*n* = 64) are shown. CNTL vs SCZ: PANSS score ≥ 90 *p* = 0.004, CNTL vs SCZ: PANSS score < 90 *p* = 0.063 (**a**). **b** The horizontal lines represent the median, and statistical differences are highlighted (Mann-Whitney *U* test), *p* < 0.05

After excluding 6 patients who were homozygous at the CAG locus, there were 147 patients with psychiatric disease (109 SCZ patients and 38 MDD patients) and 80 CNTLs for whom the degree of XCI skewing was analyzed. The ratio of XCI skewing in psychiatric patients and controls is shown in Table 1. We define the degree of skewed XCI ≥ 75:25% or ≥ 80:20% as severely skewed XCI. Using a cut-off of 75:25% for XCI skewing, SCZ patients (28.7%, *p* = 0.01) had a higher frequency of skewed XCI than CNTLs (12.5%), while MDD patients (18.4%) showed no significant difference from CNTLs, as shown in Table 1. Using a cut-off of ≥ 80:20% for XCI skewing showed similar results. Interestingly, the exaggerated XCI skewing (skewed XCI ≥ 90:10%) was predominately seen in SCZ patients (*n* = 5/35, 14.2%) with PANSS scores ≥ 90, while XCI skewing in SCZ patients (*n* = 3/64, 4.6%) who had PANSS scores < 90 did not significantly differ from that of CNTLs (*n* = 1/80, 1.25%) (Fig. 1b).

### Patient age matters in the relationship between XCI skewing and SCZ

**Fig. 2:**
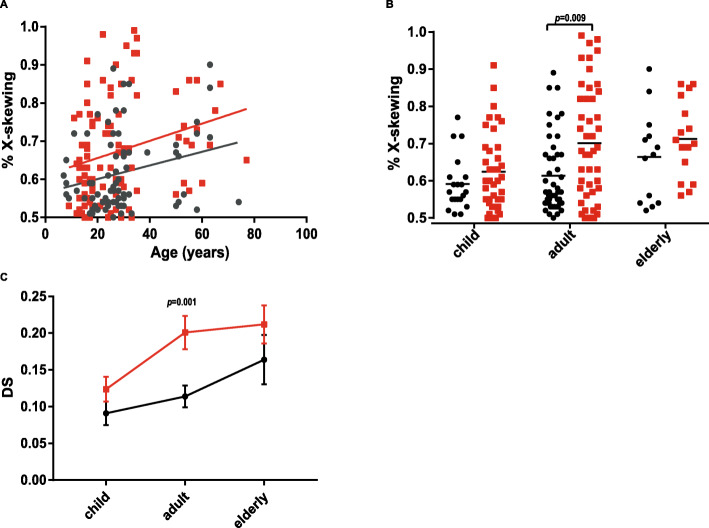
Age matters in the relationship between XCI skewing and schizophrenia. Red = SCZ, black = CNTL. **a** Scatterplot of age and the degree of XCI for CNTLs (black, *r* = 0.167, *p* = 0.138) and SCZ patients (red, *r* = 0.31, *p* = 0.002**) by Spearman’s correlation, simple linear regression (*y* = 0.002x + 0.61), and logistic regression (*p* = 0.042, OR = 1.369, 95% CI, 1.012 1.853). **b** X-skewing of child, adult, and elderly SCZ patients (red), and CNTLs (black) were compared by Mann-Whitney *U* test. Adult CNTL vs adult SCZ, *p* = 0.009, *p* < 0.0167*. **c** The degree of skewed XCI observed in adult CNTLs vs adult SCZ patients, *p* = 0.001, *p* < 0.0167* by unpaired-sample *t* test

As already reported, the frequency of XCI skewing increases with age, particularly in populations over 50-60 years of age [39]. In our study, we found that XCI skewing was significantly correlated with patient age in SCZ patients (*r* = 0.31, *p* = 0.002**) but not in CNTLs (*r* = 0.167, *p* = 0.138) (Fig. 2a). Simple linear regression analysis revealed an increase of 0.62 in skewed XCI with each 10-year increase in patient age in the SCZ group. Logistic regression analysis showed, in predicting the presence of severely skewed XCI in schizophrenia, that the OR of the patient age of schizophrenia with an increase of 10 years in patient age was 1.377 (95% CI: 1.019-1.860, *p* < 0.05). After adjusting for confounding factors, the association in schizophrenia patients (OR = 1.369, 95% CI: 1.012-1.853, *p* < 0.05) remained statistically significant. To investigate the relationship between skewed XCI and patient age, the psychiatric patients and controls were divided into a child group (≤ 18 years), an adult group (19-35 years), and an elderly group (≥ 50 years) (shown in Table 1). Using a cut-off of 75% for XCI skewing, the frequency of skewed XCI in adult SCZ patients (39%) was higher than that of adult CNTLs (12.2%, *p* = 0.005), whereas no significant differences were found between SCZ patients and CNTLs in the child and elderly groups, as shown in Table 1. For SCZ groups, the frequency of skewed XCI in the adult SCZ patients (39%) was higher than that of the child SCZ patients (15.3%) but not of the elderly SCZ patients (31%). For the CNTL group, a significant higher frequency of severely skewed XCI was found in the elderly CNTLs (23%) than in child (5%) and adult CNTLs (12.2%). Similar results were obtained using 80% as cut-off value for the definition of skewed XCI. However, for MDD groups, no difference in severely skewed XCI was found between adult and elderly MDD patients (Table 1). In addition, the degrees of skewed XCI in SCZ patients in each age group were higher than those in age-matched CNTLs, especially in the adult group (*p* = 0.002). There was a shift in the curve for the severity of skewed XCI with advanced age in the SCZ group compared with the age-matched CNTL group (Fig. 2c).

### Association between XCI skewing and clinical symptoms in SCZ

**Fig. 3:**
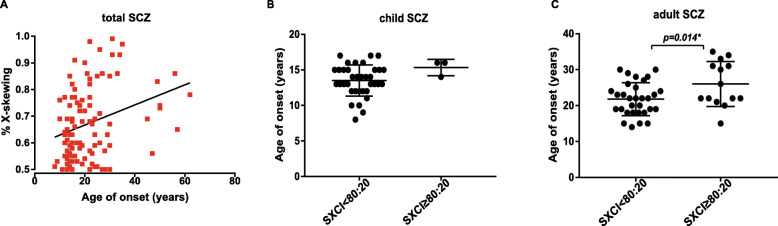
**a** Scatterplot of the age of onset and XCI skewing observed in schizophrenia patients (*r* = 0.3, *p* = 0.002* by Spearman’s correlation, simple linear regression, *y* = 0.00377*x + 0.5911, logistic regression *p* = 0.009 OR = 1.0 95% CI, 1.01-1.10). **b**, **c** We analyzed the age of onset in the child and adult groups divided by severe XCI (SXCI ≥ 80:20) vs random XCI (SXCI < 80:20): child *p* = 0.02, adult *p* = 0.014*, *p* < 0.0167* by unpaired-sample *t* test

We investigated the association between the degree of skewing and clinical features of SCZ and MDD, such as disease onset, duration, clinical symptoms, and medication. In patients with SCZ, we found a higher frequency of severely skewed XCI in the group with a PANSS score of ≥ 90 (Fig. 1b), but we did not observe a significant difference in the correlation between the degree of skewing and PANSS scores, as shown in Table 2. Psychiatric treatment had no significant effect on the XCI pattern in our study, as shown in Table 2. The relationship between patient age and the age of disease onset and the degree of skewing in SCZ showed a significant correlation, as shown in Table 2. We found that XCI skewing was significantly correlated with the age of onset in SCZ patients (*r* = 0.3, *p* = 0.002) (Fig. 3a). Logistic regression analysis showed, after adjustments for confounding factors, that the association in schizophrenia patients (OR = 1.05, 95% CI, 1.01-1.10, *p* < 0.05) was statistically significant. There was a difference between age and the age of onset (*t* = 6.62, *p* < 0.001) (supplement Figure 2). To exclude the influence of patient age, we compared the age of onset in the different SCZ age groups. Interestingly, in the child and adult groups, the age of disease onset was older in SCZ patients with skewed XCI ≥ 80:20 than in SCZ patients with skewed XCI < 80:20 (adult group *p* = 0.014) (Fig. 3c). No correlation was observed between the clinical characteristics and the degree of skewed XCI (Tables 2 and 3). Together, our data suggested that the degree of XCI skewing might play important roles in patient age and onset age, and SCZ patients with severely skewed XCI may have more severe psychiatric symptoms.

**Table 2:** The relationship between the degree of skewed XCI and clinical symptoms in SCZ

SCZ patient		Patient age (years)	Age of onset (years)	Disease duration (months)	PPANSS^†^	NPANSS^§^	Total PANSS	Medicine (mg/day)
Total patient	Coefficient	0.312	0.317	−0.053	0.1	−0.103	0.041	0.019
	Significance	0.001	0.001	0.605	0.326	0.311	0.687	0.88
Child patient	Coefficient	0.132	0.212	−0.219	0.046	−0.281	−0.061	−0.052
	Significance	0.421	0.195	0.180	0.783	0.083	0.711	0.771
Adult patient	Coefficient	0.129	0.126	−0.198	0.299	0.077	0.223	0.286
	Significance	0.391	0.402	0.193	0.046	0.614	0.142	0.236
Elderly patient	Coefficient	0.127	0.345	−0.359	0.282	0.274	0.248	0.388
	Significance	0.64	0.208	0.188	0.308	0.322	0.372	0.153

The relationship between degree of skewed XCI and clinical indicators analyzed by Spearman's correlation and Spearman's correlation was significant at *p* < 0.0167 for age group of SCZ patients.

†Positive symptoms in the PANSS

§Negative symptoms in the PANSS

**Table 3:** The relationship between the degree of skewed XCI and clinical symptoms in MDD

MDD patient		Patient age (years)	Age of onset (years)	Disease duration (months)	HAMD	Medicine (mg/day)
Total patient	Coefficient	0.123	−0.007	−0.167	0.138	−0.16
	Significance	0.451	0.966	0.323	0.415	0.33
Adult patient	Coefficient	−0.165	−0.029	−0.186	0.41	0.109
	Significant	0.462	0.896	0.408	0.058	0.688
Elderly patient	Coefficient	−0.147	−0.235	−0.385	−0.086	−0.321
	Significance	0.586	0.4	0.157	0.759	0.145

The relationship between degree of skewed XCI and clinical indicators analyzed by Spearman's correlation and Spearman's correlation was significant at *p* < 0.025 for age group of MDD patients.

†Positive symptoms in the PANSS

§Negative symptoms in the PANSS

### No genetic transmission of XCI skewing from mothers to daughters with SCZ

**Fig. 4:**
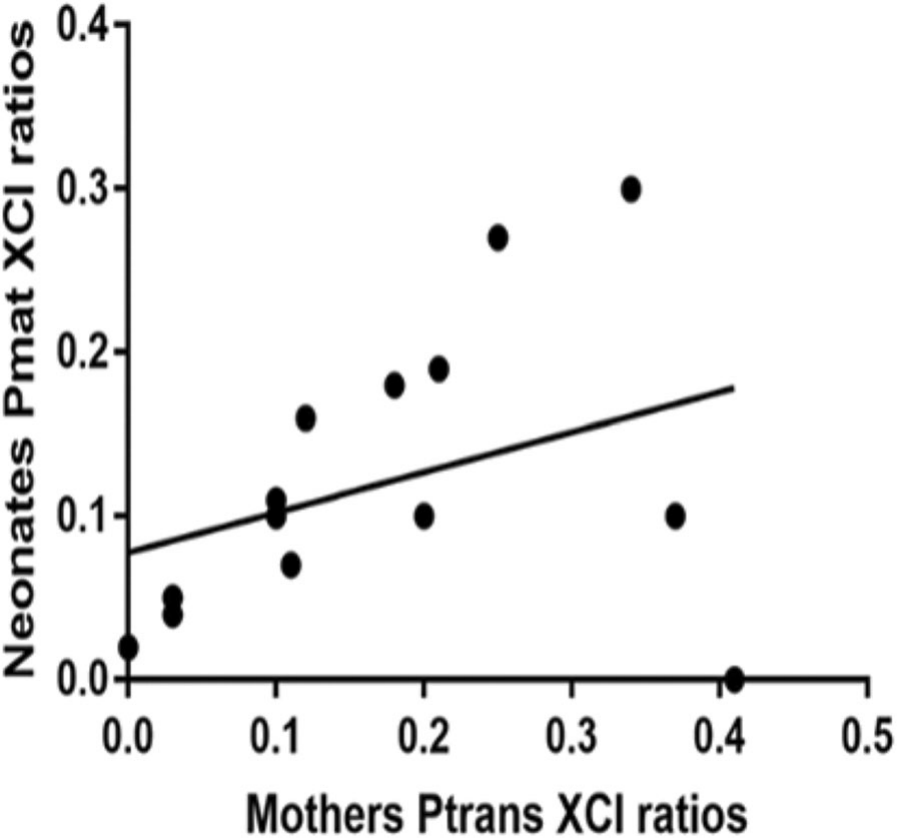
Linear regressions and correlations of mother versus child XCI ratios (*y* = 0.2448*x + 0.07787, *r*^2^ = 0.1257). Ptrans and Pmat scores were used to show the proportion of cells having the transmitted (mothers’) or the self (daughters’) allele active in mother-daughter duos (*n* = 14, *r* = 0.214, *p* = 0.355) by Spearman’s correlation

To investigate whether XCI skewing was genetically transmitted from the mother to the daughter with SCZ, we analyzed whether the SCZ patient and the patient’s mother shared a similar XCI skewing pattern. Using qualitative analysis, we examined the incidence of skewed XCI in young patients and their parents. Our data showed no difference in the frequency of skewed XCI between the SCZ patients with mothers with skewed XCI and those with mothers with nonskewed XCI (*p* = 1.0). Using a quantitative analysis by comparing the degree of skewing of the shared X chromosome in mothers and patients, we further confirmed no correlation between the groups, as shown in Fig. 4 (*r* = 0.214, *p* = 0.355). These results indicated no genetic transmission of the skewing trait. To investigate whether the XCI skewing in SCZ patients is influenced by mother or father X chromosome, we examined the genetic connect of active X chromosome between the SCZ patients with the patient’s parents. Eight of 14 (57%) SCZ patients had the maternal X and 6 of 14 patients (43%) had the paternal X as the predominating active X chromosome. These results imply that the XCI skewing in young SCZ patients was unlikely to be inherited from their parents.

## Discussion

There are many differences in clinical symptoms, the age of disease onset, and antipsychotic treatment response between male and female SCZ patients [3, 40–42]. Abnormal X chromosome function may play an essential role in the sex-related phenotype of SCZ [4]. It is unknown whether skewed XCI, as one X chromosome abnormality, might affect female SCZ patients.

In this present study, we first showed that SCZ patients had a higher frequency of severely skewed XCI than age-matched CNTLs, and the SCZ patients with severe clinical symptoms showed a higher frequency of severely skewed XCI than those who had less severe clinical symptoms (Fig. 1b). Although XCI skewing has been reported in mammalian cells for decades, the mechanisms of the XCI skewing still remain unknown. There are several hypothesized mechanisms that may result in selection against deleterious alleles [27]. For example, a study found that 7.6% of female intellectual disability patients had extreme skewing (> 90:10) to carry mutations of X-linked genes, such as *MECP2*, *DDX3X*, and *SMC1A*, which might result in selection against the cells with mutated genes carried on the active X chromosome [32]. In addition, this phenomenon also is found in some X-linked diseases, in which carriers are skewed away from the expression of the mutant allele, such as in Wiskott–Aldrich syndrome (WAS) [43]. Genetic mechanisms that may lead to XCI skewing have previously been described in mammals, such as mutations within the XIC such as *XIST* promoter mutations [44]. However, in our case, genetic transmission of XCI skewing from mother to daughter seems unlikely, since we failed to find a correlation in the degree of skewed XCI between the SCZ patients with mothers with skewed XCI and those with mothers with nonskewed XCI (Fig. 4). Another large-scale investigation of XCI skewing in 502 mother-neonate pairs also showed a similar result, which might reflect that the XCI pattern is not due to a single heritable genetic locus but rather corresponds to a complex trait to be determined [25]. Other hypothetical mechanisms for XCI skewing in humans are stochastic or age-related skewing during the developmental period. A study found that increased skewing with age was a consequence of hematopoietic stem cell senescence [45]. As the age of disease onset in SCZ patients is relatively young, whether the significant acceleration of XCI skewing in SCZ is related to any of these hypothetical mechanisms needs further investigation.

Indeed, there might be a secondary nonrandom choice of XCI, or it may occur through nongenetic mechanisms such as aging. To understand whether age is specifically linked to SCZ, we compared the XCI skewing between SCZ patients and CNTLs in three different age groups. First, we showed that the degree of skewed XCI in SCZ patients was greatly correlated with patient age and the age of disease onset (Fig. 2a, Figure 3a). Then, we observed that the level of skewing in adult SCZ patients is similar to the level in elderly CNTLs. The SCZ patients appeared to have a shift in the age-related skewed XCI curve toward the left (Fig. 2c). Our data suggested an interesting phenomenon that the average age of initial skewed XCI in SCZ patients occurred in the child group with a similar degree of skewing as seen in the adult CNTL group. The skewing of the XCI ratio seen in the blood cells of aging women is a stable biological phenomenon [46], and our data suggested that advanced aging might be one of the pathological mechanisms of SCZ in female SCZ patients. These data are supported by other studies that showed that SCZ is an accelerated-aging disease [47–50]. The hypothesis is supported in some aspects such as age-related biology markers, cognitive studies, and imaging studies [47–49]. However, most early studies on SCZ and aging are limited to elderly individuals or one age group, such as young adults. Our study included three age groups of SCZ patients and CNTLs, from children to adults and elderly individuals, which provided a more complete picture to evaluate the hypothesis.

To examine whether the level of skewed severity is associated with clinical symptoms and phenotypes of SCZ, we performed an analysis of the degree of skewed XCI and total PANSS score, positive symptoms, negative symptoms, and the age of disease onset in SCZ patients (Table 2). First, we showed more severely skewed XCI in young SCZ patients than in age-matched CNTLs, using a cut-off of 80% (Table 1). Second, we found that the severity of XCI skewing in the SCZ patients was associated with a later onset age (Fig. 3). However, we did not find a significant association between the skewing of XCI and disease duration in SCZ patients, nor did we find a similar correlation in MDD patients, as shown in Tables 2 and 3. In the same patient age group, the SCZ patients with severe skewed XCI appeared to have later age of onset than those with random or mild skewed XCI (Fig. 3c). While it is unclear whether the severe skewed XCI is also related to the typical later onset age in female SCZ than males, we did not see such an age of onset effect on XCI skewing in MDD which often with no sex difference in disease onset age. Further support from studies of X-linked diseases, which showed XCI skewing can lead to late-onset disease in patients with X-linked sideroblastic anemia, scleroderma, and common variable immune deficiency [28, 51, 52]. Although the mechanisms of skewed XCI severity in late-onset diseases were very much disease-specific, such as hematopoietic stem cell loss [53] and variable immunodeficiency [28], some shared mechanisms were also proposed as the consequence of age-related DNA mutation and gene selection [54, 55]. Because the sample size was relatively limited in the current study, the relationship between the severity of skewed XCI and disease onset age in SCZ still needs to be tested in a large-scale study in the future.

Last, we found that the SCZ patients with severe symptoms (PANSS score ≥ 90) had more extremely skewed XCI (Fig. 1b). Although there was no direct evidence of skewed XCI influencing clinical symptoms in SCZ patients, other studies have demonstrated some relationships between X chromosome inactivation and SCZ-like symptoms. For example, patients with Klinefelter syndrome, characterized by a 47,XXY chromosomal pattern, often express schizophrenia symptoms, including negative symptoms, positive symptoms, and general psychopathology, as evaluated by the PANSS, but healthy controls with a 46,XY chromosomal pattern do not [56]. This finding may be related to the changes in brain structure. Such a hypothesis has been supported by reduced gray matter volume in 47,XXY males [57], and similar reduced gray matter volume has also been found in SCZ patients with severe positive symptoms [58]. In addition, skewed XCI is also related to X-linked gene mutations, such as hemophilia A and Rett syndrome, and, in particular, when the mutated genes are located on the active X chromosome, the patients often have more severe symptoms [59–61]. Many X-linked gene polymorphisms have been associated with the symptoms of SCZ [4]. For instance, *MAOA* gene polymorphisms were positively correlated with aggressive and negative symptoms evaluated by the PANSS [62], while carriers of the high-activity allele of *MAOA* present higher scores on behavioral measures of impulsivity than carriers of the low-activity allele of *MAOA* [63]. The *MECP2* rs2734647 polymorphism might lead a lower expression level of *MECP2* and more aggressive symptoms [64]. However, whether those X-linked gene polymorphisms in SCZ are associated with the relationship between skewed XCI and PANSS scores needs to be investigated in the future.

To investigate whether the observed skewed XCI in SCZ patients was inherited from the patients’ mothers, we examined XCI skewing in 14 mother-daughter pairs in which the daughter had SCZ. We did not observe the transmission of the XCI skewing pattern from the mother to the daughter (Fig. 4). Our study, for the first time, investigated the XCI skewing in SCZ and suggested no inheritable XCI skewing in SCZ.

There were several limitations in this study. First, as XCI skewing is highly related to age, in this study, we included three age groups of SCZ patients and CNTLs to examine the disease-related XCI skewing from childhood to old age. However, we did not have age-matched MDD patients as a disease control group in this portion of the study; therefore, whether the skewed XCI in young SCZ patients is disease-specific needs to be further investigated. Second, in the current study, we examined XCI skewing only in the DNA isolated from leukocytes based on previous studies suggesting that skewed XCI in the periphery may reflect the X chromosome inactivation pattern in other tissues, including the brain [65]. In addition, it is known that systemic changes are involved in age-associated XCI, and the circulatory system and blood are involved in some of those changes [66]. The investigation of isolated DNA from multiple tissues of SCZ patients might be needed. Third, in the current study, we examined only the X chromosome inactivation pattern without examining the number of X chromosome based on previous studies demonstrating the rarity of skewed XCI in X chromosome aneuploidy samples [67]. The examination of X chromosome number for SCZ patients might be needed.

## Conclusion

To our knowledge, this is the first study to demonstrate a higher frequency of skewed XCI in female SCZ patients than in matched CNTL subjects, and the skewed XCI in SCZ patients was significantly associated with clinical psychosis symptoms. Furthermore, we found a significantly earlier onset of skewed XCI in adult SCZ patients than in age-matched CNTLs. Our findings suggested that skewed XCI might involve the disease onset as well as severity of clinical symptoms in female SCZ. The outcomes from this study provided a novel insight of the sex-specific biological mechanisms of SCZ. To support the effectiveness of XCI status as a biomarker of SCZ, further research is needed on larger female groups.

## Supplementary information

**Additional file 1: Table S1.** General demographics of subjects and clinical categories. **Figure S1.** A.B.C the distribution of age of onset in child adult elderly group, the green linear = skewed XCI ≥ 80:20. Child group: r = 0.20, p = 0.21, y = 0.01*x + 0.4787; adult group: r = 0.127, p = 0.39, y = 0.007371* + 0.53131; elderly group: r = 0.3328, p = 0.2078, y = 0.001712* + 0.6436. **Figure S2.** Compared age of onset and age by Paired Sample Test (t = 6.62, p < 0.001).

## Data Availability

The datasets used and/or analyzed during the current study are available from the corresponding author on reasonable request.

## Abbreviations

XCI: X chromosome inactivation
SCZ: Schizophrenia
CNTL: Control
PANSS: Positive and Negative Syndrome Scale
HAMD 17: 17-item Hamilton Depression Rating Scale
