# Supplementary material for: A new sex-specific underlying mechanism for female schizophrenia: accelerated skewed X chromosome inactivation
Source: Biol Sex Differ. 2020 Jul 17;11:39. doi: 10.1186/s13293-020-00315-6 (PMC7368719; doi:10.1186/s13293-020-00315-6)
Supplement: Supplementary file 1 — Additional file 1: Table S1. General demographics of subjects and clinical categories. Figure S1. A.B.C the distribution of age of onset in child adult elderly group, the green linear = skewed XCI ≥ 80:20. Child group: r = 0.20, p = 0.21, y = 0.01*x + 0.4787; adult group: r = 0.127, p = 0.39, y = 0.007371* + 0.53131; elderly group: r = 0.3328, p = 0.2078, y = 0.001712* + 0.6436. Figure S2. Compared age of onset and age by Paired Sample Test (t = 6.62, p < 0.001). [file 13293_2020_315_MOESM1_ESM.docx]

**Supplement Table 1 General demographics of subjects and clinical categories.**

| Group | SCZ | | | | MDD | | |
| --- | --- | --- | --- | --- | --- | --- | --- |
|  | Total | Child Patients | Adult Patients | Elderly Patients | Total Patients | Adult Patients | Elderly Patients |
| Patient Age (years) | 27.70±1.49 | 15.12±0.34 | 27.89±4.39 | 57.81±7.23 | 38.31±2.49 | 25.86±0.89 | 55.44±1.09 |
| Age of onset(^(^years) | 21.86±1.1 | 13.64±0.34 | 23.08±0.81 | 39.6±3.72 | 31.15±2.65 | 23.36±0.99 | 42.60±5.16 |
| Duration (months) | 56.15±8.69 | 19.71±3.13 | 43.15±5.09 | 189.8±40.26 | 41.57±9.33 | 31.81±6.63 | 55.00±20.11 |
| TPANSS | 78.48±2.34 | 86.35±2.89 | 74.6±3.42 | 69.6±7.99 | NA | NA | NA |
| PPANSS | 21.51±1.00 | 24.58±1.52 | 20.00±1.19 | 18.06±3.69 | NA | NA | NA |
| NPANSS | 19.38±0.82 | 22.30±1.10 | 18.08±1.35 | 15.66±1.59 | NA | NA | NA |
| Medicine (mg/day) | 13.42±0.84 | NA | 15.3±1.03 | 14.68±0.85 | 14.21±0.87 | 12.73±1.17 | 16.25±1.16 |
| HAMD17 | NA | NA | NA | NA | 17.28±1.31 | 22.63±0.96 | 9.93±5.74 |

Supplement Table 1 All values presented as the mean ± the SEM. CNTL healthy individuals, SCZ schizophrenic patients, MDD major depressive disorder, PANSS Positive and Negative Syndrome Scale, PPANSS positive symptoms in the PANSS, NPANSS negative symptoms in the PANSS, TPANSS total score on the PANSS, HAMD17 17-item Hamilton Depression Rating Scale.


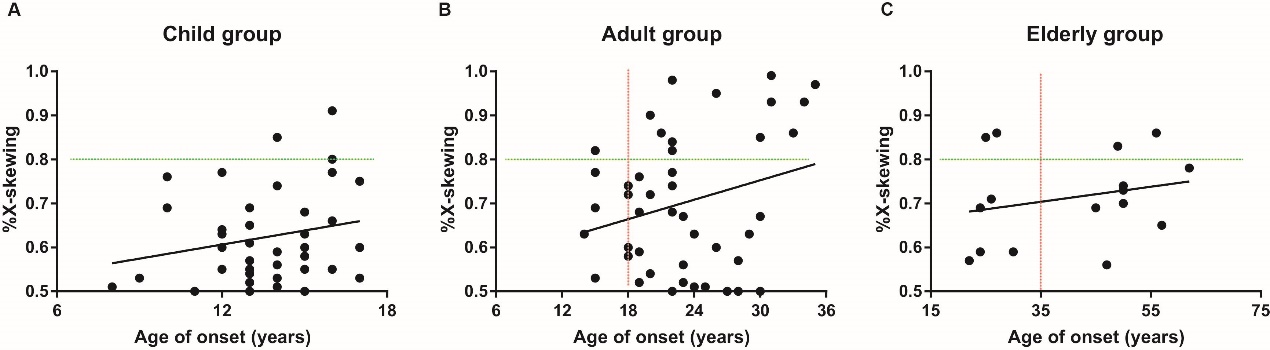


**Supplement Figure 1:** A.B.C the distribution of age of onset in child adult elderly group, the green linear =skewed XCI≥80:20. Child group: r=0.20, p=0.21, y=0.01*x+0.4787; adult group: r=0.127, p=0.39, y=0.007371*+0.53131; elderly group: r=0.3328, p=0.2078, y=0.001712*+0.6436.


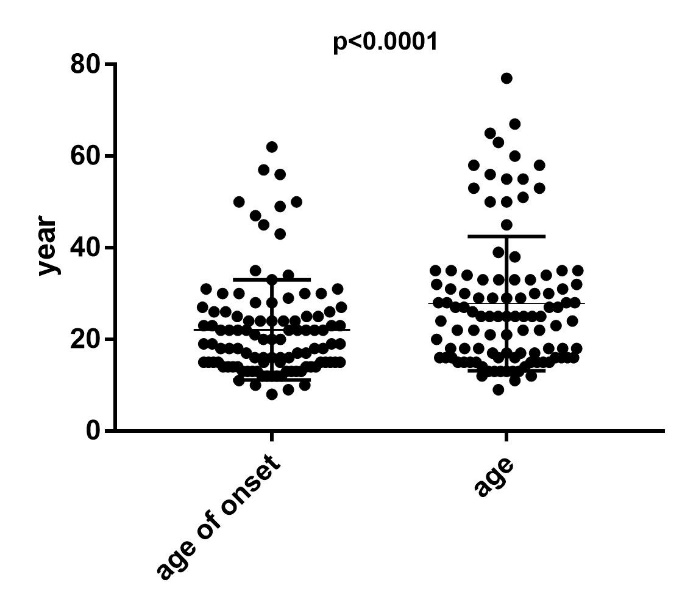


**Supplement Figure 2:** Compared age of onset and age by Paired Sample Test (t=6.62, p<0.001).
